# Acute Aortic Occlusion as an Initial Presentation of Antiphospholipid Syndrome

**DOI:** 10.31138/mjr.120423.aao

**Published:** 2023-08-30

**Authors:** Sunil Kumar Jena, Debashis Maikap, Santosh Kumar Dash, Anupam Jena, Prasanta Padhan

**Affiliations:** 1 Department of Critical Care,; 2 Department of Clinical Immunology and Rheumatology,; 3 Department of Neurology,; 4 Department of Cardiology, Kalinga Institute of Medical Sciences, KIIT University, Bhubaneswar, Odisha, India

**Keywords:** aortic occlusion, antiphospholipid syndrome, angiogram

## PRESENTATION

A 25-year-old male presented with sudden-onset severe back pain radiating down both legs, along with rapidly progressive leg weakness, paraesthesia, and cola-coloured urine with low urine output for 1 day. On examination, there was an absent pulse in both lower limbs associated with weakness. Other system examinations were normal. Laboratory values showed a haemoglobin level of 8 g/L, a leukocyte count of 20,000/cmm, and a platelet count of 4.1 lakh/cmm. There was lactic acidosis and an elevated D-dimer. The patient had raised liver enzymes, lactate dehydrogenase, and creatinine kinase, suggestive of acute rhabdomyolysis. Additionally, the patient had elevated urea (100 mg/dL) and creatinine (3.36 mg/dL) levels. Antiphospholipid workup showed raised anti-Cardiolipin IgG & IgM with a positive lupus anticoagulant test. Other thrombotic workup was negative.

**Figure 1.**
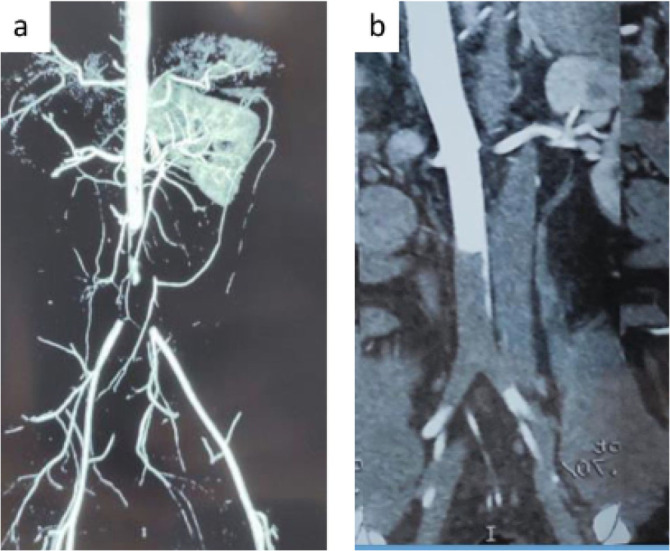
CT angiogram of aorta showing infra renal abdominal aorto-iliac thrombosis.

CECT abdomen and angiography revealed a non-enhancing thrombus in the infra-renal abdominal aorta with extension into the bilateral common iliac arteries and inferior mesenteric artery (**Figure 1**), suggestive of aortoiliac thrombosis.

The patient underwent emergency catheter-directed thrombolysis and was started on heparin, followed by oral anticoagulation. His condition improved after 7 sessions of plasmapheresis and haemodialysis. At the 3-month follow-up, he could walk without support.

## DISCUSSION

Aorto-iliac occlusive disease, also known as Leriche syndrome, is a rare and severe form of atherosclerosis that is more common in men in their sixties and is strongly associated with multi-site atherosclerosis.^1^ The potential mechanisms underlying thrombotic Leriche syndrome, which is associated with high morbidity and mortality rates, are unknown. Severe acute occlusion may result in spinal cord ischemia, resulting in neurological defects such as acute paraplegia. Our case had acute aortoiliac occlusive disease due to antiphospholipid syndrome.^2^ Antiphospholipid syndrome (APS) is defined by vascular thromboembolic events and/or pregnancy morbidity in association with antiphospholipid antibodies (aPL), which target phospholipid-binding proteins.^3^ aPL disrupts physiological haemostasis by binding to beta-2-glyco-protein I and possibly coagulation regulators, resulting in increased endothelial activation, impaired scavenging of thrombo-inflammatory mediators, and facilitated activation of the coagulation cascade.^4^ Concurrent infectious, hormonal, or traumatic stimuli (such as major surgery) are frequently required to trigger thrombosis in addition to aPL.^5^

This case highlights acute abdominal aortic occlusion is a rare presentation of antiphospholipid syndrome that necessitates rapid diagnosis and intervention.
